# General practitioners' beliefs about effectiveness and intentions to prescribe smoking cessation medications: qualitative and quantitative studies

**DOI:** 10.1186/1471-2458-6-277

**Published:** 2006-11-08

**Authors:** Florian Vogt, Sue Hall, Theresa M Marteau

**Affiliations:** 1 Health Psychology Section, Department of Psychology (at Guy's), Institute of Psychiatry, King's College London, London, UK; 2 Department of Palliative Care and Policy, School of Medicine at Guy's, King's College and St Thomas' Hospitals, King's College London, London, UK

## Abstract

**Background:**

General practitioners' (GPs) negative beliefs about nicotine dependence medications may act as barriers to prescribing them.

**Methods:**

Study1: Twenty-five GPs from 16 practices across London were interviewed in this qualitative study. Framework analysis was used to identify key themes. Study 2: A convenience sample of 367 GPs completed an internet-based survey. Path-analysis was used to examine the relations between beliefs and intentions to prescribe smoking cessation medications.

**Results:**

Study 1: Whilst nicotine replacement therapy (NRT) and bupropion were generally perceived as effective and cost-effective, the effectiveness of NRT was seen as critically dependent on behavioural support for smoking cessation. This dependence appeared to be influenced by perceptions that without support smokers would neglect psychological aspects of smoking and use NRT incorrectly. GPs perceived bupropion as dangerous and were concerned about its side-effects. Study 2: GPs' beliefs had medium (NRT, *f*^2 ^= .23) to large (bupropion, *f*^2^=.45; NRT without support, *f*^2^=.59) effects on their intentions to prescribe medications. Beliefs about effectiveness of NRT and bupropion and the perceived danger of bupropion were the key predictors of intentions to prescribe NRT and bupropion, respectively. Beliefs about neglecting psychological aspects of smoking and incorrect use had indirect effects on intentions to prescribe NRT without support, operating via beliefs about effectiveness.

**Conclusion:**

GPs vary in their beliefs about the effectiveness and safety of smoking cessation medications. Their intentions to prescribe these medications vary in line with these beliefs. Interventions aimed at increasing the likelihood with which GPs prescribe these medications may be more effective if they addressed these beliefs.

## Background

Helping people to stop smoking is one of the most effective ways of preventing premature death and reducing health inequalities [1]. In the UK, a main strategy to achieve this is to increase the number of smokers that use the NHS Stop Smoking Services (NHS-SSS) which offer free individual or group support by full-time staff trained in providing behavioural support and nicotine dependence medications, nicotine replacement therapy (NRT) and bupropion (Zyban), on prescription [1]. These medications are available on prescription to all smokers who want to stop independent of their desire to use NHS-SSS[2]. The medications and services, alone and in combination, are highly cost-effective in comparison to the great majority of medical interventions [2,3].

NRT provides a means of delivering nicotine that was formerly acquired through smoking, and thereby relieves cravings for nicotine and the symptoms of nicotine withdrawal. Meta-analyses show that quitting with NRT has an odds ratio of 1.7 compared to quitting with placebo or no treatment one year after the intervention [4]. Even when NRT is used without support (e.g. bought over the counter), its benefit is undiminished [5]. In addition to being effective, NRT is considered safe, although some negative effects of nicotine on myocardial workload mean that guidelines recommend that patients with cardiovascular conditions only use NRT after careful consideration. The effect of nicotine acquired through NRT is, however, no worse than that acquired through smoking [4].

Apart from NRT, the NHS also supports the use of bupropion as an aid for smokers who want to stop smoking. The exact mechanisms by which it facilitates smoking cessation are unknown although it is assumed to work directly on the brain pathways involved in addiction and withdrawal [4]. Meta-analyses show that quitting with bupropion has an odds ratio of 2.4 compared to quitting with placebo one year after the intervention [4]. Bupropion increases the risk of epileptic seizures, and is therefore contraindicated in patients who are at risk for seizures. Seizures occur in about 1 in 1000 patients using bupropion. This relative risk is only an approximate estimate because no direct comparative studies have been conducted. A recent case-series analysis concludes that despite statistically non-significant findings there is probably an increased risk of seizures associated with the use of bupropion, with a relative incidence of seizures of 3.62 (95% CI 0.87 to 15.09) [6].

Smoking cessation guidelines [2] and the new NICE Public Health Intervention Guidance [7] recommend that general practitioners (GPs) advise all smokers to stop smoking and provide medications and/or refer smokers who are motivated to NHS-SSS. The guidance recommends that GPs offer the behavioural support (e.g. NHS-SSS) first. If smokers are not interested they should be offered prescriptions for NRT or bupropion [7]. It is estimated, however, that smoking cessation advice is given in only 20%–30% of UK primary care consultations with smokers [8]. One study estimated that only 6% of GPs have referred smokers to smokers' clinics and 41% to nurses trained in smoking cessation in the previous month [9]. Another study showed that less than 5% of smokers were advised about NRT by their GP [10]. Studying GP self-reports, 80% of patient requests for NRT and 59% of patient requests for bupropion were found to be honoured by GPs [11].

Failure to implement evidence-based guidelines is not restricted to smoking cessation [12,13]. Interventions to increase adherence to guidelines using a wide-variety of methods including incentives, prompts/reminders, and education have had mixed results and there is no clear evidence to favour any particular strategy [13]. Critiques of this large literature highlight that most interventions lack explicit rationales or theoretical bases [12,14]. A first step to developing an intervention to increase the frequency with which GPs prescribe smoking cessation medications to smokers who are motivated is to identify the factors that may influence this clinical practice. Motivational theories propose that motivation is a proximal determinant of behaviour [15-18]. Interventions should therefore target factors that determine motivation. Examples of such determinants include beliefs about the consequences of a behaviour and attitudes towards performing the behaviour.

A systematic review showed that while the majority of GPs and family physicians do not hold negative beliefs and attitudes towards discussing smoking cessation with their patients, a sizeable minority do [19]. Discussing smoking cessation was perceived as too time consuming by 42%. Thirty-eight percent believed it was ineffective and 22% reported lacking confidence in their ability to discuss smoking cessation with their patients. Few studies, however, have assessed beliefs about smoking cessation medications. Those that have, report that about 20% of GPs believed that assisting smokers by directing them to use NRT is inappropriate, unfeasible, and not cost-effective [9,20]. Fewer believe it is ineffective [9,20] and 30% would give NRT a low priority in the drug budget [11]. Many GPs are concerned about the side-effects of bupropion (69%) and a third would give bupropion a low priority in the drug budget [11]. In general, little is known about GPs perceptions of smoking cessation medications. In particular, the quantitative nature of the available evidence limits the insight they can provide to fully understand GPs' perceptions about them. For example, whilst this research showed that sizable proportions of GPs believed that assisting smokers with NRT was inappropriate [20], it is unknown why GPs believed this. Similarly, it is unclear why around 30% of GPs would give NRT and bupropion a low priority in the drug budget [11]. Although the proportions of GPs who believe that NRT is ineffective amounts to only around one-tenth [9,20], this finding is surprising given the clear evidence of the effectiveness of NRT [4]. Identifying the specific beliefs that underlie some of the broad categories of beliefs is important in designing interventions to change GPs' beliefs.

A major feature of qualitative methods is their ability to describe and display phenomena as experienced by the study population in fine detail and in the study participants' own terms. They therefore offer the opportunity to 'unpack' phenomena, to see what they are about or what lies behind them [21]. Qualitative research also allows associations that occur in people's thinking or acting to be identified [21]. The aim of Study 1 is to provide an in-depth understanding of the basis for GPs' beliefs about smoking cessation medication, using qualitative methods. This is followed by a second study aimed at describing the prevalence of these beliefs and the strength of their association with intentions to recommend smoking cessation medications.

## Methods

### Study 1

#### Participants

Twenty-five GPs, whose practices are part of the Medical Research Council General Practice Research Framework (MRC GPRF), were interviewed. Ten of the GPs were female and fifteen were male (age range 27 to 60).

#### The interview

Interviews were conducted by a researcher trained in qualitative interview techniques (FV). A semi-structured interview schedule was used covering topics related to discussing smoking with smokers, NRT, bupropion, and behavioural support for smoking cessation (NHS clinics and local services). Emphasis was given to criteria used for deciding whether or not treatments are introduced into health care [22], that is, their effectiveness and cost-effectiveness. The interview schedule was piloted with three GPs from a London general practice, and refined where appropriate. Interviews were audio-taped and transcribed verbatim.

#### Data Analysis

The data were managed using NVivo software for qualitative data analysis and analysed using the framework method [21]. Framework analysis has five stages: familiarisation, identifying a thematic framework, indexing, charting, mapping and interpretation. Details of the analysis process are shown in Table 1. Internal validity was established through the 'constant comparative method' [23], involving constant and repeated checking of the interpretation of the data, inherent in the five stages of framework analysis [21]. In addition, the thematic analysis was supported and verified by two experienced researchers by ascertaining consensus in the interpretation. The internal validity was enhanced by displaying quotations to supplement the analysis where quotations explicitly document linkages or explanations. To address external validity, 'methods triangulation' [21], which relies on generating data by another method, was used. Study 2 serves for triangulation and validation of the themes that emerged from Study 1. Only themes regarding smoking cessation medications are shown in the results section. Themes relating to smoking cessation services are presented elsewhere [24].

#### Procedure

The MRC GPRF sent invitation letters to all 128 practices in the framework in the Greater London area. Of these, 16 agreed to take part in the study. From these, 30 GPs agreed to be interviewed. Five did not have time to be interviewed in the limited data collection phase of the study. GPs were interviewed in their practices. At the start of the interview they were assured of anonymity. The interviews lasted between 10 and 30 minutes, depending on how much each participant had to say. After the interviews, all participants were offered £20 book tokens as compensation for their time.

### Study 2

#### Participants

Three-hundred and sixty-seven general practitioners completed the survey. All were users of an internet-based medical information service provider. At the time of study, 4743 UK GPs (~12% of UK GPs) were registered users of this information services provider. Of the 381 GPs who were invited to participate, only fourteen (4%) did not complete the questionnaire. Seven (2%) declined and seven (2%) deferred completion. Respondents were generally representative of the general population of GPs based on the Department of Health Statistics for General Medical Practitioners with a bias towards men responding (about 60% of GPs in the UK are male) (Table 2). This bias reflects the profile of GPs that are registered with the service provider.

#### Procedure

A questionnaire was presented to any member of the service provider registered as a general practitioner upon accessing the service during four days in November 2004. When members log on to visit the Web site, they must give their General Medical Council number. Software checks this information as well as a list of available questionnaires. Members then have three choices: (i) complete the questionnaire immediately, (ii) defer completion to another time, or (iii) refuse completion. The service provider carries out regular profit and not-for-profit survey research among its members and has been successfully used for academic purposes [25]. Rewards are offered to GPs if they respond to questionnaires on a regular basis.

#### Measures

The questionnaire content was designed on the basis of the interviews with the 25 GPs as reported in Study 1. It was piloted with 22 GPs using the same methods as the main study.

##### Intention

Intention is defined as the expressed motivation to perform some behaviour or achieve some goal [26]. Two items were used to measure intentions using the stem, "Thinking about the next month, do you intend to..." and "Thinking about the next month, how likely is it that you will...". The stems were followed by "...prescribe NRT to all motivated smokers" (α = .756), "...prescribe bupropion to all motivated smokers" (α = .866). Only one item was used to measure intentions to prescribe NRT without behavioural support due to space restrictions on the questionnaire, using the "...intend to..." stem with "...prescribe NRT to all motivated smokers even without behavioural support". The "...intend to..." stem had a response range from 1 (definitely do not) to 7 (definitely do). The "...how likely..." stem had a response range from 1 (very unlikely) to 7 (very likely).

##### Beliefs

Perceived effectiveness was measured using the stem "...is effective at helping motivated smokers to stop smoking" and perceived cost-effectiveness using the stem "...is effective enough to justify its cost" [9]. Both perceived effectiveness, and perceived cost-effectiveness were preceded by "NRT...", and "Bupropion...". Perceived effectiveness of NRT without behavioural support was measured with the item "NRT without behavioural support is ineffective at helping motivated smokers to stop smoking". Perceived incorrect use and perceived neglect of psychological aspects of smoking were measured regarding NRT without behavioural support using the items "NRT without behavioural support will result in NRT being used incorrectly" and "NRT without behavioural support neglects psychological aspects of smoking". Concerns about the side-effects of bupropion was measured with the item "I am concerned about the side effects of bupropion" based on a similar measure used in another study [11]. Perceived danger of bupropion was measured using two items "Bupropion is dangerous for my patients" and "Bupropion unnecessarily endangers the lives of what for the most part, are healthy smokers" (α = .821). All beliefs were measured using a response range from 1 (strongly disagree) to 7 (strongly agree).

#### Path analysis

Path analysis is a concise way to organise causal thinking and is an extension of the statistical method of multiple linear regression [27]. In addition to multiple regression analysis, path analysis assembles the antecedents of the outcome variable into a structure of presumed causal relationships. Given such a presupposed causal model, path analysis estimates the magnitude of the linkages between the variables. The language of the method invokes causality in speaking of "causal" paths and effects but this language assumes that the model is correct [27]. Causal pathways operating via two or more variables indicate indirect effects. Indirect effects are very similar to mediated effects. A mediator is a variable *"to the extent that it accounts for the relation between the predictor and the criterion" *[28, p. 1176]. To establish the significance of indirect effects operating through two or more variables covariance structure software is considered superior [29]. The covariance structure software AMOS [30] was used to calculate the estimate path coefficients using a bootstrapping method (2000 bootstrap samples were used) [31]. The path models were based on Study 1. All beliefs were initially considered as direct predictors of intentions (non significant paths were removed from the models). The Squared Multiple Correlation (SMC) of each outcome variable was calculated. The SMC is the equivalent to an R-squared (R^2^) in linear regression [32]. Effect sizes were calculated for the variance explained in intentions using *f*^2 ^= R^2^/(1-R^2^) [33]. According to Cohen an *f*^2 ^value of 0.02 is small, an *f*^2 ^value of 0.15 is medium, and an *f*^2 ^value of 0.35 is large.

#### Data preparation

Prior to analysis, the variables were examined for accuracy of data entry, missing values, and violations of multivariate assumptions and passed all required tests (some variables were not normally distributed but bootstrapping is unaffected by this). There were few missing data, well below 5% in all instances. Missing values were replaced with the mean responses of the variable.

## Results

### Study 1

#### Nicotine Replacement Therapy

GPs acknowledged that NRT had a positive impact on smokers' chances of stopping smoking even though most smokers would fail with NRT. Beliefs that contributed to this perception included the belief that NRT would help with the physical addiction to nicotine and in particular with nicotine cravings. In particular forms, such as delivered using an inhaler, NRT was seen as helping with behavioural aspects of stopping smoking by, for example, giving smokers something to do with their hands and something to focus on generally.

*'I think for a lot of patients you're breaking the habit, so you're not just treating their nicotine withdrawal, so if you're using nicotine replacement, giving them an inhalator might help them reduce the habit of having to have something to do with their hands (GP 19)'*.

NRT was also perceived as offering psychological help by clearly marking the beginning of a cessation attempt and giving it more importance.

*'Nicotine replacement is often a step in their lives, a threshold that they are definitely doing it, rather than just having a day off, two days off, and then going back to it (GP 8)'*.

In GPs' views, NRT also provided smokers with a sense of hope, which was considered as possibly the sole reason why NRT had an impact on quit rates. That NRT might work entirely on the basis of a placebo rather then having an active effective component was however not necessarily seen negatively.

*'I think a lot of patients that I give it to do find it's given them a lot of benefit. Personally, I think it's largely in the mind; I wonder, but if it's a panacea then fine as well (GP 18)'*.

Based on the belief that it helped smokers to stop smoking, NRT was mostly seen as effective enough to justify its cost. It would prevent future smoking-related illnesses and as a result reduce future health care costs. In addition, the cost of NRT was considered small, which was helped through its use being confined to a brief period.

Even though GPs were generally positive about NRT, a theme emerged that NRT was perceived as largely ineffective unless smokers also received behavioural support for smoking cessation. When describing sources of such behavioural support GPs were making reference to nurses working at the practice or smokers' clinics operated by the primary care trust. Contributing to this sense of dependence on behavioural support was a concern that smokers would simply relapse without addressing psychological aspects of smoking, such as reasons why patients smoked, when they smoked, and why they wanted to give up. Another concern was that smokers would transfer their nicotine dependence from smoking to using NRT unless they received support and guidance on how to use it, and how they should cease using it over time. There was also a sense that smokers who did not want to use behavioural support in addition to NRT were not entirely committed to stopping smoking and were therefore less likely to stop smoking.

*'It's a help, but I wouldn't say it's the total therapy. I think these people need a bit of counselling, they need to change their lifestyles. ... So yes, you know, in a certain way nicotine does help, but there needs to be an adjuvant therapy added to it (GP 9)'*.

As a result of this perceived lack of effectiveness in the absence of behavioural support, some GPs said they would only prescribe NRT for smokers who accepted some form of behavioural support, either on a one-to-one or a group basis.

*'We've got the nurse running a cessation clinic and we don't prescribe any of those products unless they make a commitment to come along to that (GP 13)'*.

The risks associated with NRT were seen as negligible, even though some negative impacts on the cardiovascular system and concerns about the abuse potential of NRT were mentioned.

*'Nicotine replacement therapy is completely safe (GP 2)'*.

#### Bupropion

The key theme that emerged about bupropion was GPs' concerns about its side-effects. Concerns were mostly related to epileptic convulsions and death. Awareness of these side-effects came from media reports and experiences with patients. It was acknowledged however that deaths were rare. As a result of the concerns about the side-effects, GPs were reluctant to prescribe bupropion and perceived it only as a second-line treatment after NRT.

*'I tend to say nicotine replacement first before Zyban, I don't quite know why. ... I think that might be my own sort of prejudice about Zyban because it's had some bad press. ... I think there has been some deaths, hasn't there, with Zyban. So I think that's probably why I'm a bit more cautious with it. Second line Zyban, I think, even though I know it shouldn't be, but I think that's because of my preconceived ideas that I've got about it, that it is potentially dangerous, isn't it, even though the numbers are miniscule (GP 7)'*.

Some therefore only prescribed it for smokers who had unsuccessfully used NRT to stop smoking. GPs also tried to limit the number of smokers that would use bupropion by not offering it to their patients, but instead waiting for smokers to request it. Some also actively discouraged smokers from using bupropion.

*'If you come to see me and say that, "I would really like to stop smoking", I don't go here's some Zyban. I will sort of discuss the different options and alternatives and I might mention Zyban but I usually actually leave it up to the patient to say, "Oh I've heard there's something called Zyban can you tell me a bit about that". And I have to be pushed quite hard to prescribe Zyban (GP 2)'*.

Some had stopped prescribing it altogether.

*'I've stopped prescribing it completely since its negative publicity came out because of the side-effects that were recorded (GP 6)'*.

In spite of concerns about the side-effects, it was acknowledged that bupropion could help smokers to stop smoking. It was mentioned that bupropion would reduce the cravings for cigarettes and that it was particularly helpful for heavy smokers. Akin to perceptions about the effectiveness of NRT, GPs recognised that many smokers would still fail even if they used bupropion. Low compliance due to side-effects was seen as limiting the effectiveness of bupropion, attributed to smokers discontinuing the treatment.

*'Half of the patients have successfully quit the smoking and they swear by that it has reduced their craving and things like that and they have been helped to stop the smoking. There's a quarter of the patients who had side effects like sleeplessness etc, and had to give up. The other quarter probably haven't been really successful because the craving is still strong (GP 12)'*.

Based on the belief that it helped smokers to stop smoking, bupropion was mostly seen as being effective enough to justify its cost. It would prevent smoking-related morbidity leading to substantial cost-savings. This view was supported by the perception that bupropion was helpful for heavy smokers who were also most likely to incur large health-care costs in the future. The positive perception of cost-effectiveness was further supported by the recognition that bupropion was only used for a short period and that the monetary cost for this treatment period was quite small.

*'I'm not really sure how expensive it is but you only give it for 2 months so it can't be that expensive and again I think if it creates one long-term non-smoker then it probably paid for the other 99 people that you've given 2 months worth and then chucked it in the bin (GP 2)'*.

Some, however, also regarded bupropion as relatively expensive, at least in relation to NRT.

#### Summary of results (Study 1)

Study 1 shows that GPs are broadly positive about NRT, but believe that it is largely ineffective if offered without behavioural support. Underlying this perception of ineffectiveness of NRT if used without additional support were the perceptions that the use of NRT without behavioural support would neglect the psychological aspects of smoking and that it would be misused. In addition, bupropion was seen as having too many side-effects to be used as a first line treatment.

### Study 2

#### Prevalence of beliefs and intentions

Whereas around 79% of GPs agreed that NRT was effective, only 27% agreed that NRT was effective if used without behavioural support for smoking cessation (Table 3). Furthermore, just 64% agreed that bupropion was effective. While 64% agreed that NRT was cost-effective, fewer than 50% agreed that bupropion was cost-effective. More than 66% expressed concern about the side-effects of bupropion and 21% believed bupropion was dangerous. Reflecting these beliefs, most GPs intended to prescribe NRT to all motivated smokers, although only a minority intended to prescribe NRT without support, or bupropion.

#### Path analysis for intentions to prescribe NRT

**Figure 1:**
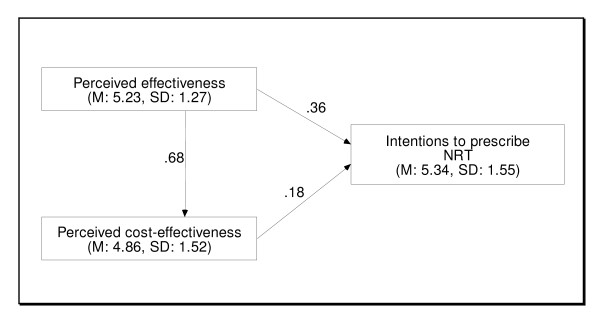
NRT path model; unstandardised coefficients, M = mean, SD = standard deviation.

'Perceived effectiveness' and 'perceived cost-effectiveness' had direct effects on 'intentions to prescribe NRT' (Table 4, Figure 1). 'Perceived effectiveness' also had a direct effect on 'perceived cost-effectiveness'. 'Perceived effectiveness' had an indirect effect on 'intentions to prescribe NRT' operating through 'perceived cost-effectiveness'. Adding up the direct and indirect effects, 'perceived effectiveness' (B = .48) had the largest total effect on 'intentions to prescribe NRT', followed by 'perceived cost-effectiveness' (B = .18).

#### Path analysis for intentions to prescribe NRT without support

**Figure 2:**
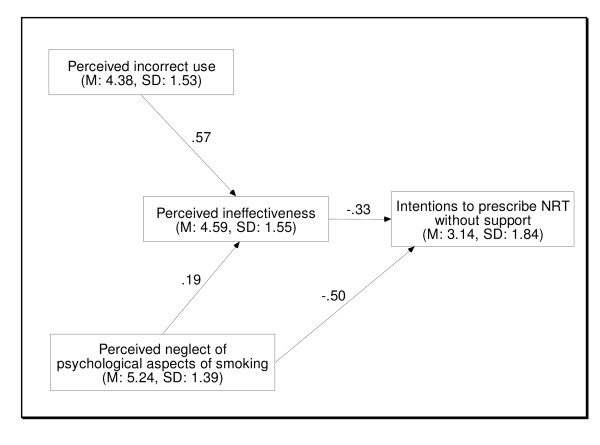
NRT without support path model; unstandardised coefficients, M = mean, SD = standard deviation.

'Perceived ineffectiveness' and 'perceived neglect of psychological aspects of smoking' had direct effects on 'intentions to prescribe NRT without behavioural support' (Table 5, Figure 2). 'Perceived incorrect use' and 'perceived neglect of psychological aspects of smoking' had direct effects on 'perceived ineffectiveness'. 'Perceived incorrect use' and 'perceived neglect of psychological aspects of smoking' had indirect effects on 'intentions to prescribe NRT without support' operating through 'perceived ineffectiveness'. Adding up the direct and indirect effects, 'perceived neglect of psychological aspects of smoking' (B = -.56) had the largest total effect on intentions to prescribe NRT without support, followed by 'NRT without support is ineffective' (B = -.33) and 'NRT without support will be used incorrectly' (B = -.19).

#### Path analysis for intentions to prescribe bupropion

**Figure 3:**
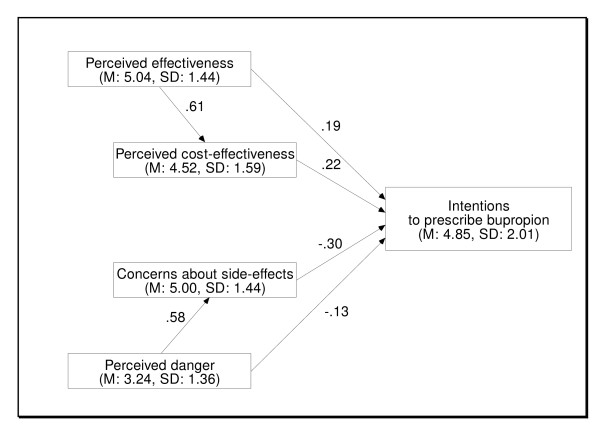
Bupropion path model; unstandardised coefficients, M = mean, SD = standard deviation.

'Perceived effectiveness', 'perceived cost-effectiveness', 'concerns about side-effects', and 'perceived danger' had direct effects on 'intentions to prescribe bupropion' (Table 6, Figure 3). 'Perceived effectiveness' also had a direct effect on 'perceived cost-effectiveness' and 'perceived danger' also had a direct effect on 'concerns about side-effects'. 'Perceived effectiveness' had an indirect effect on 'intentions to prescribe bupropion' operating through 'perceived cost-effectiveness'. 'Perceived danger' had an indirect effect on 'intentions to prescribe bupropion' operating through 'concerns about side-effects'. Adding up the direct and indirect effects, 'perceived effectiveness' (B = .32) had the largest total effect on 'intentions to prescribe bupropion', followed by the perceptions that 'perceived danger' (B = -.31), 'concerns about side-effects' (B = -.30), and 'perceived cost-effectiveness' (B = .22).

#### Summary of results (Study 2)

Study 2 showed that while the majority of GPs perceived smoking cessation medications as effective, a sizable proportion did not. In addition, only a minority believed that NRT without additional support for smokers was effective. GPs were divided about whether NHS smoking cessation medications were cost effective. Path analysis suggested that these beliefs were strongly related to GPs' intentions to prescribe smoking cessation medications. Many GPs associated serious side-effects with bupropion which was negatively associated with GPs intentions to prescribe bupropion.

## Discussion

### Beliefs about effectiveness

The proportion of GPs that perceived NRT as ineffective in the current study confirms findings of previous studies conducted among English and Welsh GPs [9,20]. To date, the prevalence of beliefs about the effectiveness of bupropion and NRT without support have not been estimated. GPs in Study 1 and more than 50% of GPs in Study 2 perceived NRT without support as ineffective. These beliefs appear at odds with meta-analyses which show that NRT is effective independent of the intensity of additional support [5]. Whilst NRT is effective on its own, combining it with behavioural support is believed to lead to a higher quit rates than each on its own [2-4], although good evidence for this is lacking.

The results from both our quantitative and qualitative research suggested that two beliefs underlie the belief that NRT was ineffective without behavioural support. The first is that without behavioural support smokers will fail to address the psychological aspects of smoking (e.g. that smokers do not plan in advance how to deal with cravings). The second belief is that NRT without behavioural support will be used incorrectly (e.g. smokers may use it in addition to smoking). These two beliefs may be important targets for interventions aimed at changing GPs' beliefs about the effectiveness of NRT without support. Interventions may also need to address how smokers are educated about using NRT. It was expected that the effect of perceptions about neglecting psychological aspects of smoking when NRT is used without behavioural support, on intentions to prescribe NRT without support, would operate entirely through beliefs about the effectiveness of NRT if used without support in Study 2. The remaining direct effect may be related to the way in which GPs conceptualised the effectiveness of NRT without support.

The results of Study 1 suggested that GPs were reluctant to prescribe NRT without behavioural support because they believed that it is largely ineffective without it. Study 2 confirmed these findings showing that the more GPs believed that NRT without support was ineffective the less likely they were to prescribe it. Other studies have also found that GPs believed that NRT should not be prescribed if smokers do not want additional support [34]. However, we believe that the current study is the first to highlight the importance of perceived effectiveness as an explanation for GPs' reluctance to prescribe NRT without behavioural support. In contrast, the NICE Public Health Guideline and the recent White Paper are clear in that smokers should be offered a prescription for NRT regardless of whether or not they also want intensive behavioural support for smoking cessation [1,7]. Study 2 also showed that the more GPs believed that NRT and bupropion were ineffective, the less likely they were to report prescribing them.

A question that arises, though, is how GPs interpret effectiveness. They may interpret it (i) with reference to whether smokers are helped at all, or (ii) with reference to the clinical impact provided (e.g. low Number Needed to Treat (NNT) [35]). Whilst a negative response to the former can be interpreted as caused by lack of knowledge or rejection of the evidence, the latter interpretation may reflect GPs' evaluations of the perceived clinical impact informed either by personal experience, the published evidence or both. Given the large proportion of smokers that will fail to stop despite using the most effective treatments, such an assessment of clinical impact could be considered accurate. Future research needs to address how GPs conceptualise effectiveness. Combined treatments (including behavioural support and nicotine dependence medications) have produced a NNT of 50 to save one life-year [36]. This compares favourably with other medical interventions. However, even if GPs' beliefs could be seen as accurate, beliefs that smoking cessation medications are ineffective should arguably not interfere with smokers' chances of being offered them.

### Beliefs about cost-effectiveness

Sixty-four percent of GPs believed that NRT was cost-effective. In 2001, only 47% reported that they believed NRT was cost-effective [9]. However, fewer than half of GPs believed that bupropion was cost-effective. Beliefs about cost-effectiveness had a direct effect on intentions to prescribe NRT and bupropion. These findings, illustrating the importance of beliefs about the cost-effectiveness, build upon the findings from a recent study showing that GPs who believed that NRT or bupropion were effective enough to make the NHS pay for them also believed that NRT and bupropion should be available on prescription [11]. The current study is the first to show the importance of GPs' beliefs about cost-effectiveness with regards to GPs' motivation to make smoking cessation medications available for smokers. Interestingly, in Study 1, GPs talked about cost-effectiveness in terms of cost-savings, rather than cost-per-life-year (quality-adjusted) saved, as practiced by institutions [4]. It appears therefore that GPs awareness of how cost-effectiveness judgments are made uses a different framework to that used by policymakers. In summary, GPs appear to consider cost in relation to effectiveness, in keeping with the recommendation that they do so by agencies such as the National Institute for Clinical Excellence (NICE) [22]. Unfortunately, however, GPs' judgments do not match the accepted evidence, with many judging some of the most cost-effective of all health care interventions, that is, smoking cessation medications, as not cost-effective.

### Concerns about the side-effects of bupropion

GPs in Study 1 perceived that the adverse effects of bupropion were too great to justify its use as a routine nicotine dependence treatment. These findings are in accord with the results of a recent cross-sectional survey which found that GPs' concerns about the side-effects of bupropion were correlated to GPs' not fulfilling prescription requests for bupropion [11]. The quantitative study provides additional support by being the first to show a link between anticipated side-effects, perceptions of danger and reduced motivation to prescribe bupropion. This suggests that there may be a causal influence of concerns about side-effects leading to GPs being less likely to offer bupropion, as previously suggested [11].

In Study 2 a similar proportion of GPs (66%) to that reported in a previous study using a random sample of UK GPs (69%) [11] were concerned about the side-effects of bupropion. One in five GPs believed that bupropion is dangerous. In the UK there has been a considerable amount of negative media response to the safety of bupropion prompting a response from the Medicines Control Agency (MCA) to highlight the relative safety of bupropion [37]. Recent figures show that prescriptions for bupropion in the UK have fallen dramatically to approximately one-third of the level in 2000/01 [38]. This may reflect the negative media reports and the consequent reluctance of GPs to prescribe bupropion. The Royal College of Physicians considers the risk of seizures to be small and no more than that incurred by the use of other antidepressants [39]. Some GPs may be strongly influenced by short-term harms as opposed to the long-term benefit of stopping smoking regardless of their likelihoods. Seizures, one of the most severe side-effects, occur in less than 0.1% of users [4], whilst one in every two smokers will die prematurely if they continue to smoke [39]. Whether it is possible or advisable to alter GPs beliefs is a question of debate.

### Strengths and limitations

#### Study 1

A strength of this study is that the qualitative method revealed previously not recorded beliefs and behavioural patterns. The study has two limitations. First, financial and time constraints limited the length of the interviews. This limited the depth with which beliefs could be explored. Second, the sample of the current study was restricted to GPs working in MRC GPRF-registered practices. Although MRC GPRF practices are representative in terms of the distribution of partnership size compared to the distribution of practices in the UK, these practices volunteer to dedicate some of their efforts to enhancing evidence-based medicine. GPs working in such practices and those that participated in the current study are thus likely to have a more favourable view of evidence-based medicine than GPs working in practices that do not. The findings may therefore reflect a more favourable perspective on smoking cessation medications and services than may have been found in a more representative sample of UK GPs. In addition, only a small proportion of GPs from GPRF practices volunteered to participate, possibly making the sample unrepresentative of GPRF practices on the whole. Those who volunteered may have been more favourable towards smoking cessation medications and services than those GPs who did not.

#### Study 2

The predictive ability of the models used to explain intentions to recommend smoking cessation services compares very favourably to the effects sizes achieved in predicting intentions that are generally reported with motivational theories (e.g. Theory of Planned Behaviour) [40]. In addition, effect sizes in the current study are based upon specific beliefs instead of more abstract and general concepts such as the attitudes included in the Theory of Planned Behaviour. By using specific beliefs the current studies suggest specific intervention targets to increase GP prescription of smoking cessation medications. There are several limitations. Alternative models could have been constructed from the observed data and these may have provided different insights. Furthermore, due to financial limitations only a small number of items could be included in the survey. This limited (i) the range of beliefs that could be assessed (e.g. perceived cost, beliefs about the consequences of behaviour), and (ii) the number of items used to assess beliefs. The latter prohibited removing measurement error and thereby decreased the power of the analysis [40]. Finally, the sample was not randomly selected from the population of GPs and although the sample broadly reflected demographic characteristics of GPs' in the UK, GPs responding via the Internet might not be representative of the wider population of GPs. GPs responding via the internet may have different attitudes towards nicotine dependence medications than the wider population of GPs. On the current topic, evidence for the generalisability of results is provided by the observation that concerns about bupropion matched those of a recent randomly selected sample of GPs [11].  Furthermore this method should not affect the relationships between beliefs and intention to prescribe nicotine dependence medications. The current sample had a male bias when compared with the national population of GPs, reflecting the profile of GPs that are registered with the service provider. However beliefs and intentions of male and female GPs did not differ in the current study (analysis not shown).

## Conclusion

The two studies reported here identified substantial reservations amongst GPs about NRT and bupropion. In particular, these concern the effectiveness of NRT without additional support, the safety of bupropion and the cost-effectiveness of NRT and bupropion. Beliefs about the effectiveness and concerns about safety had large effects on intentions to prescribe smoking cessation medications. Path analysis provided clues about beliefs underlying perceptions of ineffectiveness of NRT without additional behaviour support. Addressing these beliefs may be important if GPs are to encourage more smokers to use smoking cessation medications.

## Competing interests

The author(s) declare that they have no competing interests.

## Authors' contributions

FV, SH, and TMM are responsible for the design of the studies; FV performed the interviews; FV was responsible for the acquisition and analysis of the data; SH and TMM participated in the analysis and interpretation of results; FV, SH, and TMM contributed to the conception and design of the paper; FV drafted the paper; SH and TMM critically revised the paper. All authors read and approved the final version of manuscript.

## Pre-publication history

The pre-publication history for this paper can be accessed here:



## Figures and Tables

**Table 1:** Process of data analysis

FV reads interview transcripts and generates *codes *for beliefs reported by GPs about intervening with smokers and about smoking cessation medications and services.
FV reads and compares the codes to identify *themes *within these: the principal beliefs GPs reported about intervening with smokers and about smoking cessation medications and services.
Data relating to each *theme *are assembled. FV, SH, and TM independently read these and discuss definitions of *themes *and the data within these. During this process some definitions are altered and some data re-coded.
FV re-codes all transcripts for these *themes*.
FV begins coding for *sub-themes *within themes: more detailed variations in GPs' thinking within themes. *Sub-themes *include GPs' explanations for reasons behind preferring practices to smoking cessation clinics.
The process of coding for sub-themes includes building a *framework *containing themes and sub-themes. This process includes shifting themes to sub-themes and vice versa.
The framework is arranged in tables using text segments to represent the themes in order to facilitate understanding of the data
FV, SH, and TM study the tables to gain an understanding of themes and sub-themes and decide on main issues. Disagreements are discussed and amendments made where appropriate.

**Table 2:** Demographic and background details of participants

**Characteristic**	**Levels**	**N**	**%**
Gender	Female	60	16.3%
	Male	307	83.7%

Decade of qualification	1960s	11	3%
	1970s	122	33.2%
	1980s	146	39.8%
	1990s	86	23.4%
	2000s	2	0.5%

Commitment	Full time	323	88%
	Pat time	44	12%

Country of training	UK	327	89.1%
	Other	40	10.9%

Geographical location	London	32	8.7%
	South East	63	17.2%
	South West	40	10.9%
	West Midlands	41	11.2%
	Eastern	46	12.5%
	Trent	44	12%
	North West	56	15.3%
	Northern and Yorkshire	45	12.3%

Presence of trained practice nurse	No	89	24.3%
	Yes	277	75.5%
	missing	1	0.3%

**Table 3:** Prevalence of beliefs and intentions

	Proportion			
**Beliefs (mean, *SD*)**	**Agree (> 4)**	**Neutral (4)**	**Disagree (< 4)**	**Missing**
NRT is effective. (5.23, 1.27)	79.4% (291)	11.7% (43)	8.9% (33)	-
NRT without behavioural support is effective. ^a ^(4.59, 1.55)	26.5% (96)	20.7% (76)	52.5% (190)	1.4% (5)
NRT is cost-effective. (4.86, 1.52)	64.1% (234)	20.0% (73)	15.8% (58)	0.5% (2)
Bupropion is effective. (5.04, 1.44)	65.4% (240)	14.2% (52)	20.4% (75)	-
Bupropion is cost-effective. (4.52,1.59)	45.9% (166)	24.9% (90)	29.3% (106)	1.4% (5)
Concerned about so-effects of bupropion. (3.86, 1.60)	66.2% (242)	18.3% (67)	15.5% (57)	0.3% (1)
Bupropion is dangerous. (5.00, 1.44)	20.7% (76)	13.6% (50)	65.7% (241)	-
				
**Intentions (mean, SD)**	**Intend (> 4)**	**Neutral (4)**	**Do not intend (< 4)**	**Missing**

Intention to prescribe NRT. (5.34, 1.55)	79.8% (293)	4.9% (18)	15.3% (56)	-
Intention to prescribe NRT without support. (3.14, 1.84)	29.5% (108)	9.8% (36)	60.7% (222)	0.3% (1)
Intention to prescribe bupropion. (4.85, 2.01)	20.7% (76)	9.5% (35)	69.8% (256)	-

Notes: ^a ^reversed scale.

**Table 4:** NRT: direct, indirect, and total effects on beliefs and intentions

**Outcome variable**	**Predictor variable**	**Effects ^a^**		
		**Direct**	**Indirect**	**Total**
Intention to prescribe NRT SMC = 0.19, 95% CI: .12 to .27, p < .01. *f*^2 ^= 0.23	Perceived effectiveness	.36***	.12***	.48***
	
	Perceived cost-effectiveness	.18 ***	-	.18***

Perceived cost-effectiveness SMC = 0.33, 95% CI: .23 to .43, p < .01.	Perceived effectiveness	.68***	-	.68***

Notes: ^**a **^unstandardised coefficients, **p < 0.05, ***p < 0.01.

**Table 5:** NRT without support: direct, indirect, and total effects on beliefs and intentions

**Outcome variable**	**Predictor variable**	**Effects ^a^**		
		**Direct**	**Indirect**	**Total**
Intention to prescribe NRT without support SMC = 0.31, 95% CI: .23 to .39, p < .01. *f*^2 ^= 0.45	Perceived ineffectiveness (without support)	-.33***	-	-.33***
	
	Perceived neglect of psychological aspects of smoking	-.50***	-.06***	-.56***
	
	Perceived incorrect use	-	-.19***	-.19***

Perceived ineffectiveness (without support)	Perceived incorrect use	.57***	-	.57***
	
SMC = 0.43, 95% CI: .36 to .52, p < .01.	Perceived neglect of psychological aspects of smoking	.19***	-	.19***

Notes: ^**a **^unstandardised coefficients, **p < 0.05, ***p < 0.01.

**Table 6:** Bupropion: direct, indirect, and total effects on beliefs and intentions

**Outcome variable**	**Predictor variable**	**Effects ^a^**		
		**Direct**	**Indirect**	**Total**
Intention to prescribe bupropion SMC = 0.37, 95% CI: .29 to .45, p < .01. *f*^2 ^= 0.59	Perceived effectiveness	.19***	.14***	.32***
	
	Perceived cost-effectiveness	.22***	-	.22***
	
	Concerns about side-effects	-.30***	-	-.30***
	
	Perceived danger	-.13**	-17***	-.31**

Perceived cost-effectiveness SMC = 0.33, 95% CI: .22 to .44, p < .01.	Perceived effectiveness	.61***	-	.61***

Concerns about side-effects SMC = 0.30, 95% CI: .22 to .38, p < .01.	Perceived danger	.58***	-	.58***

Notes: ^**a **^unstandardised coefficients, **p < 0.05, ***p < 0.01.
